# Cytotoxicity and Genotoxicity of Resin-Based Dental Composites Modified with Quaternary Ammonium Salts

**DOI:** 10.3390/jfb16120459

**Published:** 2025-12-09

**Authors:** Izabela Szymczak-Pajor, Maja Zalega, Joanna Nowak, Agnieszka Śliwińska, Katarzyna Woźniak, Kinga Bociong

**Affiliations:** 1 Department of Nucleic Acid Biochemistry, Medical University of Lodz, 251 Pomorska Str., 92-213 Lodz, Poland; izabela.szymczak@umed.lodz.pl (I.S.-P.); agnieszka.sliwinska@umed.lodz.pl (A.Ś.); 2 Department of General Dentistry, Medical University of Lodz, 251 Pomorska Str., 92-213 Lodz, Poland; maja.zalega@student.umed.lodz.pl; 3 University Laboratory of Materials Research, Medical University of Lodz, 251 Pomorska Str., 92-213 Lodz, Poland; joanna.nowak.1@umed.lodz.pl; 4 Department of Molecular Genetics, Faculty of Biology and Environmental Protection, University of Lodz, 141/143 Pomorska Str., 90-236 Lodz, Poland; katarzyna.wozniak@biol.uni.lodz.pl

**Keywords:** resin-based composites (RBCs), quaternary ammonium salts (QAS), cytotoxicity, genotoxicity

## Abstract

The primary reason of dental restoration failure is the recurrence of caries, driving research to incorporate quaternary ammonium salts (QASs) into resin-based composites (RBCs). Given the prolonged contact of these materials with oral tissue, this in vitro study assessed the biocompatibility (cytotoxicity and genotoxicity) profiles of experimental RBCs modified with cetyltrimethylammonium bromide (CTAB) and dimethyldioctadecylammonium bromide (DODAB), using two restorative materials: an unmodified–experimental composite, KE, and Flow-Art (FA) as comparative standards. The primarily novelty of this study is the direct comparison of the cellular safety profiles of CTAB vs. DODAB when incorporated into RBCs. Human fibroblast BJ cells were exposed to composite eluates for 24 h, and cell viability (MTT assay), the percentage of apoptotic and necrotic cells (the Annexin V/Propidium Iodide (PI) flow cytometry method), and DNA damage (the alkaline comet assay) were quantified. Among the compounds evaluated, only CTAB caused a significant, dose-dependent decrease in BJ cell viability, primarily by inducing late apoptosis or necrosis. Cell viability was severely reduced, dropping by 84% at 2 wt% CTAB (*p* < 0.001) compared to control. Consistent with this effect, CTAB also induced a dose-dependent increase in DNA damage. In contrast, the DODAB-modified composites, along with the KE and FA controls, exhibited non-cytotoxic and non-genotoxic profiles across all tested concentrations. This innovative comparative assessment highlights that DODAB exhibits superior cellular safety, offering vital guidance to prioritize its use for developing safe and effective next-generation antibacterial dental composites. Conversely, CTAB is precluded for clinical use at these concentrations due to its observed toxicity.

## 1. Introduction

The global burden of dental caries remains substantial, with approximately 39% of the world population suffering from untreated lesions, a figure that has risen considerably [1,2]. Caries is nearly ubiquitous in adults (>90%) and highly prevalent in adolescents (>60%) [3]. The primary treatment involves restoration with photopolymerizable resin-based composite (RBCs). RBCs are the material of choice for dental restorations owing to their superior aesthetic and mechanical properties [4,5,6]. These materials are complex polymers [7] composed of an organic resin matrix (typically 15–50 wt%), a photo-initiator, and silanized reinforcing filler particles [5,8,9,10,11].

Secondary caries is the leading reason for replacement of 57% to 88% of existing RBCs restorations [12,13]. Its etiology is primarily linked to the formation of a marginal gap caused by factors like polymerization shrinkage, material porosities or poor adaptation that facilities the colonization of pathogenic bacteria [14,15,16]. Bacterial adhesion, subsequent biofilm accumulation, and acidic metabolite production induce demineralization of adjacent tooth structure and contribute to composite degradation [17,18]. Residual caries from incomplete removal of infected dental tissue poses a further challenge [19]. Recognizing that cariogenic bacteria drive disease progression, the World Health Organization (WHO) emphasizes the critical need to incorporate antibacterial additives into RBCs [20,21,22,23]. To address these issue, research has focused on developing new RBCs with intrinsic antibacterial properties [24,25]. Two main antibacterial strategies are currently explored: 1. Antibacterial agent release: This provides high local antimicrobial concentrations but often suffers from short-lived efficacy and can negatively compromise material properties. 2. Contact-dependent strategy: This offers prolonged antibacterial activity with less adverse mechanical effects, through its antimicrobial action is often weaker and susceptible to reduction via surface biofouling [24]. In recent years, attention has focused on incorporating antimicrobial nanoparticles and submicrometer-size particles (e.g., zinc oxide [26], zinc-doped mesoporous silica nanoparticles [27], titanium dioxide [28], silver [29], silver sodium hydrogen zirconium phosphate [30], cellulose nanocrystal/zinc oxide nanohybrids [31], chlorhexidine release system [32,33], essential oils [34], or polymerizable compounds such as imidazole and chitosan particles/nanocomposites [35,36]. Despite varying success in laboratory evaluations, frequently reported drawbacks include compromised aesthetic properties [37], cytotoxicity [38], and reduced mechanical integrity [39,40]. Notably, studies indicate that incorporating quaternary ammonium salts (QASs) into RBCs holds promising potential for caries prevention due to their ability to inhibit bacterial growth [41,42].

The effective antimicrobial properties of quaternary ammonium compounds QACs stem from their structure as cationic-headed, hydrophobic-tailed polymerizable methacrylates [43,44,45]. While the precise mechanism of QAS antibacterial action remains under investigation, published evidence suggests that the positively charged QAS molecules disrupt the negatively charged bacterial cell membrane. This membrane perturbation induces cytoplasmic leakage and, specifically the efflux of essential cellular components, ultimately leading to pathogen death [23,43,44,45,46,47]. Given these properties, QACs are widely incorporated into various dental materials, as detailed in Table 1.

CTAB is frequently used for preparing materials such as mesoporous silica nanocomposites [57]. Its antibacterial action, particularly against *Escherichia coli*, is documented to involve bacterial cell membrane disruption and the induction of superoxide stress, causing intracellular component leakage [58,59,60]. CTAB forms micelles, and its high mobility generally grants it superior microbicidal activity in liquid solution as it efficiently penetrates and lyses cells [60,61,62,63].

DODAB is a synthetic lipid that self-assembles into stable cationic membranes (bilayer vesicles) [64,65]. The membrane’s stability and ability to interact with negatively charged substrates are attributed to its small, positively charged cationic “head”. Additionally, DODAB functions as an effective flocculating agent. Mechanistically, both DODAB and CTAB induce bacterial cell death by modifying the cell surface charge and causing membrane destabilization [64,65]. Against fungi like *Candida albicans*, DODAB may be more potent, achieving the necessary cell viability reduction at a significant lower concentrations than CTAB [46]. When incorporated into materials, their actions diverge: DODAB is highly desirable for non-leaching, permanent antimicrobial surfaces because its double-chain structure keeps it fixed, killing microbes upon contact [63,66]. CTAB, however, often kills by leaching out of the material into the surrounding environment [60,61]. Thus, CTAB is often better for bulk disinfection, while DODAB is superior for creating durable, contact-killing surfaces.

Due to the established antibacterial efficacy of CTAB and DODAB, their incorporation presents a compelling strategy for functional modification of RBCs. However, because dental biomaterials maintain long-term direct and indirect contact with oral tissues (gingival, pulp), rigorous assessment of their biocompatibility and potential toxicity is paramount for safe clinical translation [10,67,68,69]. An ideal biomaterial must demonstrate chemical stability, robust biocompatibility, and sensory neutrality [10,67,68,69]. Biocompatibility requirements, formalized by the American Dental Association (ADA) and the Federation Dentaire Internationale (FDI) [70] and globally mandated [71], necessitate evaluating the material’s resistance to the complex oral environment (saliva, bacteria, temperature, and mechanical wear) [67]. A comprehensive assessment of biocompatibility requires multiple endpoints, including in vitro cytotoxicity, genotoxicity/mutagenicity assays, and in vivo sensitization tests [72]. Published studies indicate that RBCs release substantial amounts of uncured free monomers, implicated in the induction of oxidative stress, cytotoxicity, and genotoxicity [73]. Therefore, characterizing the cytotoxic and genotoxic properties of new RBC constituents is imperative before clinical use. The present in vitro study aimed to evaluate the biocompatibility (cytotoxicity and genotoxicity) of experimental RBCs incorporating CTAB and DODAB by comparing them to two restorative materials: an unmodified—experimental composite, KE and Flow-Art (FA), utilizing MTT, FC and Comet assays. The primary novelty of this study lies in the direct comparative assessment of the cellular safety profiles of CTAB- vs. DODAB-modified RBCs when incorporated into the composite matrix. We hypothesized that DODAB-modified composites would show lower cytotoxicity and genotoxicity than CTAB-modified ones.

## 2. Materials and Methods

### 2.1. Reagents and Chemicals

The dental composite resin matrix was formulated using the following components:10 wt% triethylene glycol dimethacrylate (TEGDMA) (Sigma-Aldrich, Saint Louis, MO, USA), 40 wt% diurethane dimethacrylate (UDMA) (Sigma-Aldrich, Saint Louis, MO, USA), 40 wt% bisphenol A glycerolate dimethacrylate (bis-GMA) (Sigma-Aldrich, Saint Louis, MO, USA), and 10 wt% 2-hydroxyethyl methacrylate (HEMA) (Sigma-Aldrich, Saint Louis, MO, USA). The incorporation of HEMA into the composite composition was optimized based on previously published data from our laboratory. This inclusion was necessary to achieve a strategic balance between enhancing the final mechanical properties and minimizing the resultant polymerization shrinkage stress [74]. 0.1 wt% of butylated hydroxytoluene (BHT) (Sigma-Aldrich, Saint Louis, MO, USA) was employed as the inhibitor of photopolymerization, 0.9 wt% of 2-(dimethylamine)ethyl methacrylate (DMAEMA) (Sigma-Aldrich, Saint Louis, MO, USA) as the co-initiator and 0.4 wt% of camphorquinone (CQ) (Sigma-Aldrich, Saint Louis, MO, USA) as the photoinitiator. Following 0.5, 1.5, and 2.0 wt% of QASs: cetyltrimethylammonium bromide (CTAB) (Sigma-Aldrich, Saint Louis, MO, USA) or dimethyldioctadecylammonium bromide (DODAB) (Sigma-Aldrich, Saint Louis, MO, USA) were added to the resin matrix. Arsil silica (Zakłady Chemiczne Rudniki S.A., Rudniki, Poland) was used as the inorganic filler and was silanized with 3-methacrylooxypropyltri-methoxysilane (γ-MPTS, Unisil Sp. z o.o., Tarnów, Poland). This silanization process was conducted following the established protocol described by Kleczewska et al. [75]. The prepared silanized Arsil silica was then manually incorporated into the resin matrix using an agate mortar to yield an experimental composite with a 45 wt% filler content. Both the unmodified and QAS-modified experimental composites were photo-cured. Samples, covered with laboratory slides, were cured for 20 s using THE CURE TC-01 polymerization lamp (SPRING; Norristown, PA, USA) with a power of 1200 mW/cm^2^ at a 1.5 mm thickness of the material. Samples of commercial composite Flow-Art (FA) (Arkona, Niemce, Poland) were prepared strictly according to the manufacturer’s instructions. From each composite material, three samples were prepared for each independent study (or assay).

### 2.2. Cell Culture, Preparation of Eluate and Cell Treatment

All experiments were performed using the BJ cells (human fibroblast cell line, ATCC^®^ CRL2522™), which was obtained from the American Type Culture Collection (ATCC, Manassas, VA, USA). Cells were maintained as a monolayer in Eagle’s Minimum Essential Medium (EMEM) supplemented with 10% fetal bovine serum, 4 mM L-glutamine and 50 IU/mL penicillin/streptomycin (Gibco, Life Technologies, Carlsbad, CA, USA). Cells were grown under standard conditions: 37 °C in a humidified atmosphere of 5% CO_2_ and 95% air, in accordance with the manufacturer’s guidelines. For all experiments, cells were utilized during their logarithmic growth phase (passages three to six). Cells were detached using a trypsin-EDTA solution, and viable cells were counted via Trypan blue staining.

To prepare the test eluates, individual samples of each investigated composite were placed into separate Eppendorf tubes. Each tube received 1 mL of complete EMEM medium and was subsequently incubated for 24 h at 37 °C. Following incubation, the resulting eluates were centrifuged at 2000 rpm for 5 min. The obtained supernatants were then collected and utilized for subsequent cytotoxicity and genotoxicity assays. Eluates were prepared in accordance with the specifications outlined in the international standard ISO 10993-12 (Biological evaluation of medical devices—Part 12: Sample preparation and reference materials) [72].

### 2.3. Cell Viability Determination (Cytotoxicity Analysis)—MTT Test

The cytotoxic effects of composite eluates on BJ cell viability were quantified using the MTT (3-(4,5 dime-thyl-thiazol-2-yl)-2, 5-diphenyl tetrazolium bromide) assay. The MTT assay relies on the capacity of viable, metabolically active cells to enzymatically reduce the water-soluble, yellow tetrazolium dye into purple formazan crystals. BJ cells were seeded into 96-well plates at a density of 5000 cells/well and incubated overnight to achieve the logarithmic growth phase. Cells were subsequently treated for 24 h with a total volume, consisting of 50 µL of fresh complete EMEM medium and 50 µL of prepared test eluates. The tested eluates were derived from composites containing CTAB (0.5; 1; 2 wt%), DODAB (0.5; 1; 2 wt%), the unmodified control composite (KE), and the commercial composite FA. Subsequent to the cellular incubation phase, 20 µL of MTT reagent (5 g/L concentration) was introduced into every well. Following a 4 h reaction period, the culture medium was removed by aspiration. The resulting formazan precipitate was then solubilized via the addition of 100 µL of dimethyl sulfoxide (DMSO) per well. The absorbance was quantified at a wavelength of 570 nm utilizing a microplate reader (LMG Biotech, Ortenberg, Germany). Cell viability was calculated as the percentage of the untreated control cells (set to 100%) and was based on the mean values obtained from three independent BJ cell culture experiments.

### 2.4. Apoptosis Detection—Flow Cytometry

Apoptosis was quantified using the Annexin V and FITC kit (BD Biosciences, San Diego, CA, USA) according to the manufacturer’s protocol. BJ cells were seeded into 6-well plates at a density of 1 × 10^6^ cells/well and incubated overnight until they reached the logarithmic growth phase. Treatment was initiated by adding 1500 µL of fresh complete EMEM medium and 1500 µL of the prepared composite eluates, followed by a 24 h incubation period. Following exposure, cells were detached by trypsinization, washed twice with phosphate-buffered saline (PBS), and pelleted by centrifugation. The collected cell pellets were resuspended in 1000 µL of a flow cytometry binding buffer. A 100 µL aliquot of this cell suspension (1 × 10^5^ cells) was stained with 5 µL of a 1:1 mixture of FITC Annexin V and propidium iodide (PI). After gentle vortexing, the mixture was incubated for 15 min at room temperature in the dark. Finally, an additional 400 µL of binding buffer was added to the cell suspension. The samples were then analyzed immediately using a flow cytometer (FACSCalibur, BD Biosciences, San Diego, CA, USA). The flow cytometry analysis utilized a sequential gating strategy to ensure accurate quantification of the target single-cell population. The initial step involved defining Gate A on the Forward Scatter (FSC-A) versus Side Scatter (SSC-A) plot, which isolated the population based on morphologically coherent cells (size and granularity) and excluded cellular debris. The subsequent singlet exclusion gate was applied by plotting FSC-Area (FSC-A) versus FSC-Width (FSC-W) to effectively eliminate cell aggregates, ensuring that only individual cells (singlets) proceeded to the final analysis. A total of 20,000 singlet events were acquired for quantitative analysis. Data acquisition and subsequent analysis were performed using Kaluza Analysis 21 Software (Beckman Coulter, Brea, CA, USA). The final dot plots illustrate the distribution of cells based on staining with FITC-Annexin V and PI, characterizing the cellular viability status across four distinct quadrants: the live cells (FITC Annexin V+, PI−; lower left quadrant; C−−), in early (FITC Annexin V+, PI−; lower right quadrant; C+−); late apoptotic-necrotic stages (FITC Annexin V+, PI+; upper right quadrant; C++) and dead cells (FITC Annexin V−, PI+; C−+).

### 2.5. DNA Damage Assessment (Genotoxicity Analysis)—Alkaline Comet Assay

The alkaline comet assay was performed to evaluate the genotoxic effect of the tested compounds, adapting the methodology of Singh et al. [76] as previously modified [77,78]. This technique detects single- and double-strand DNA breaks, and alkali-labile sites. BJ cells were seeded at a density of 5 × 10^4^/well in 12-well plates and incubated overnight. Cells were subsequently treated for 24 h with 500 µL of fresh complete EMEM medium and 500 µL of prepared eluates. Following the designated treatment interval, cells were harvested using standard trypsinization and washed. twice with cold PBS. The collected cells were then immediately resuspended in 0.75% low melting point agarose. This cell suspension was subsequently layered onto microscope slides previously coated with 0.5% normal melting point agarose. The slides were subjected to lysis for 1 h at 4 °C in a buffer containing 2.5 M NaCl, 100 mM EDTA, 1% TritonX100, and 10 mM Tris (pH 10). DNA un-winding was achieved by incubation in unwinding buffer (300 mM NaOH, 1 mM EDTA, pH > 13) for 20 min. Electrophoresis followed at 0.73 V/cm (28 mA) for 20 min. After drying and washing, slides were stained with 2 mg/mL 4′,6-diamidino-2-phenylindole dihydrochloride (DAPI) for 30 min in the dark. Comets were examined at 200× magnification using a fluorescence microscope (Nikon, Tokyo, Japan). Quantification of DNA damage was performed using LuciaComet v. 4.51 analysis software (Laboratory Imaging, Prague, Czech Republic), with the final result expressed as the mean percentage of DNA in the comet tail from 50 randomly analyzed cells per sample.

### 2.6. Statistical Analysis

All statistical analyses were performed using GraphPad Prism 6.0 (San Diego, CA, USA). Preliminary testing for the normality of data distribution was conducted using the Shapiro–Wilk test. The null hypothesis of normality was rejected if the resulting *p*-value was <0.05, indicating a non-normally distributed dataset. Differences between two groups were assessed using either the Student’s *t*-test or the Mann–Whitney U-test. If the data was normally distributed, the Student’s *t*-test was used. If the data was not normally distributed, the Mann–Whitney U-test was employed. For analysis involving three or more groups, the assumption of homogeneity of variances was also evaluated using Levene’s test, in addition to assessing data distribution. Differences among three or more groups were evaluated using the Analysis of Variance (ANOVA) with Tukey’s multiple comparison test or the non-parametric Kruskal–Wallis test with Dunn’s multiple comparison test. ANOVA with Tukey’s multiple comparison test was employed only if the data satisfied the assumption of normality (Shapiro–Wilk test, *p* ≥ 0.05) and homogeneity of variances. The Kruskal–Wallis test with Dunn’s multiple comparison test was utilized when the data failed to meet the assumption of normality (Shapiro–Wilk test, *p* < 0.05) or when the homogeneity of variance was violated. Data are presented as the mean ± standard deviation (SD) from three independent experiments. A *p*-value < 0.05 was considered to indicate statistical significance.

## 3. Results

### 3.1. The Cytotoxicity of Tested Dental Composites

**Figure 1:**
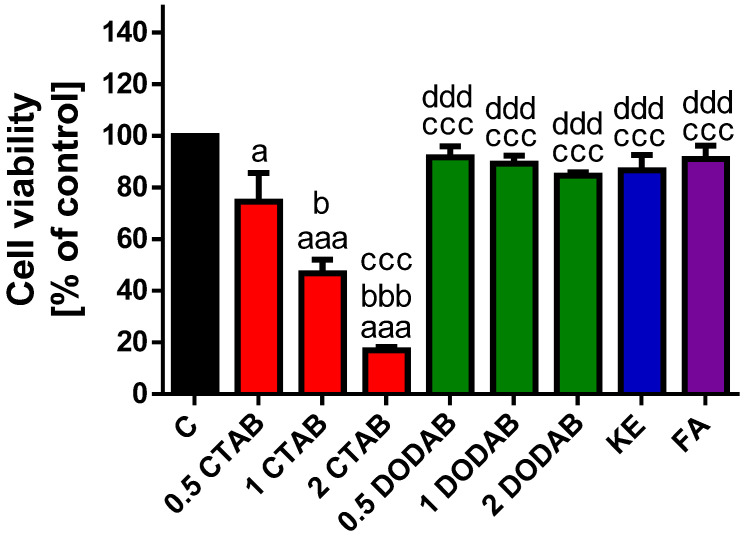
Effect of composite eluates on BJ cell viability. Cell viability was assessed using MTT assay after 24 h exposure to eluates derived from the tested composites. Treatments included eluates of composites modified with CTAB (0.5, 1, and 2 wt%; red bars), DODAB (0.5, 1, and 2 wt%; green bars), the unmodified control composite, KE (blue bar) and the commercial composite, FA (violet bar). Data are expressed as the percentage of the untreated control cells (set to 100%), and represent the mean ±standard deviation (SD) from three independent experiments. ^a^ *p* < 0.05; ^aaa^ *p* < 0.001 vs. Control (non-treated cells). ^b^ *p* < 0.05; ^bbb^ *p* < 0.001 vs. 0.5% CTAB. ^ccc^ *p* < 0.001 vs. 1% CTAB. ^ddd^ *p* < 0.001 vs. 2% CTAB.

The effect of the tested dental composites (Figure 1 and Supplementary Table S1) on the viability of BJ cells was evaluated using the MTT assay. Exposure to the eluates derived from CTAB-modified composite resulted in a significant and dose-dependent decrease in BJ cell viability (0.5 wt% CTAB vs. 1 wt% CTAB, *p* < 0.05; 0.5 wt% CTAB vs. 2 wt% CTAB, *p* < 0.001; 1 wt% CTAB vs. 2 wt% CTAB, *p* < 0.001). Cell viability was reduced by approximately 26% at 0.5 wt% CTAB (*p* < 0.05), 54% at 1 wt% CTAB (*p* < 0.001), and 84% at 2 wt% CTAB (*p* < 0.001). In contrast, the cytotoxicity effect of the eluates derived from DODAB-modified composite was markedly lower. Although a dose-dependent trend was observed, the reduction in cell viability was statistically non-significant, reaching a maximum decrease of approximately 16% at the highest (2 wt% DODAB) concentration. The eluates of the unmodified control composite, KE and the commercial composite FA showed minimal, non-significant cytotoxicity, reducing cell viability by approximately 14% and 9%, respectively. Cell viability was also significantly decreased in response to CTAB eluates (*p* < 0.001) when compared to eluates of DODAB, KE and FA.

To sum up, these results indicate that CTAB possessed the most potent cytotoxic properties among the tested materials.

### 3.2. Apoptotic Potential of Tested Dental Composites on BJ Cells

**Figure 2:**
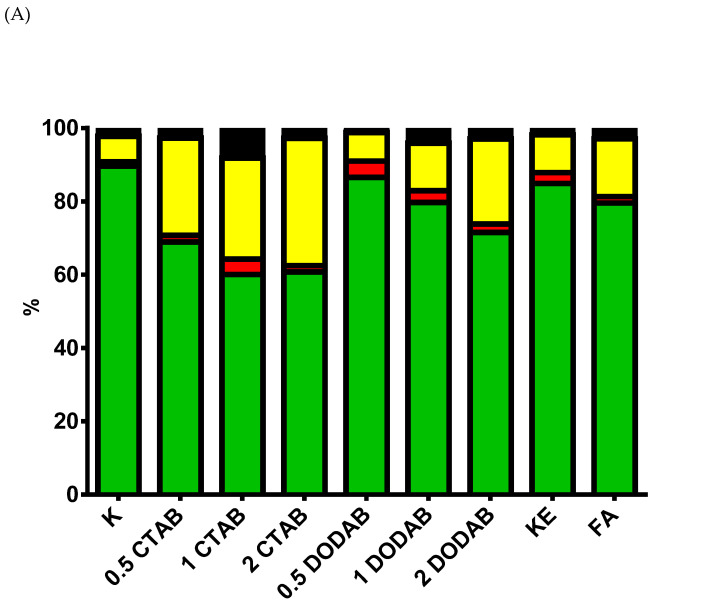

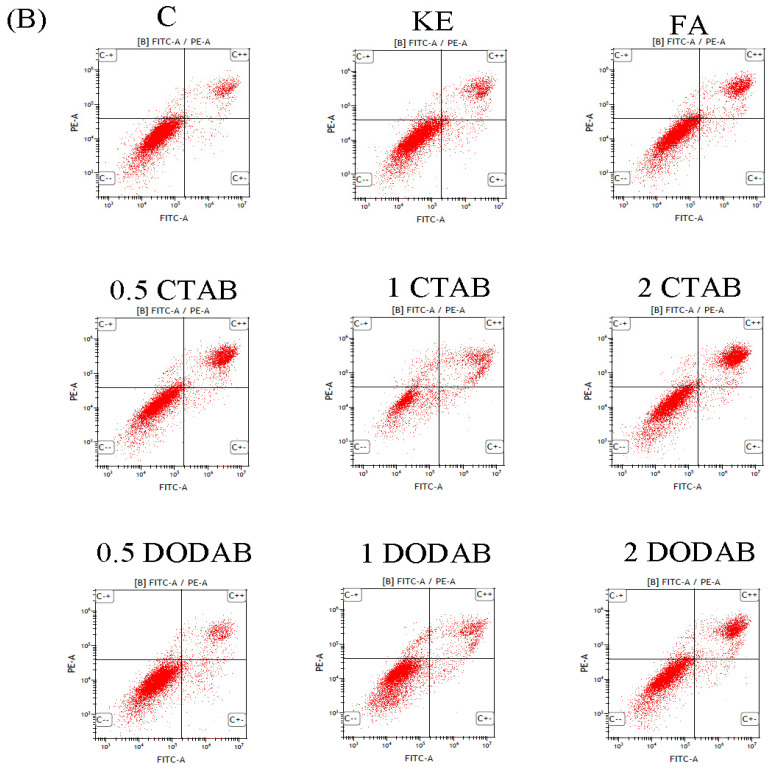
Induction of apoptosis in BJ cells by composite eluates. (**A**) The panel illustrates the percentage of cells in the following states: live cells (green bars), early apoptosis (red bars), late apoptosis/necrosis (yellow bars), and dead cells (black bars). (**B**) Dot plot graphs representing the live cells (FITC Annexin V+, PI−; lower left quadrant; C−−), in early (FITC Annexin V+, PI−; lower right quadrant; C+−); late apoptotic-necrotic stages (FITC Annexin V+, PI+; upper right quadrant; C++) and dead cells (FITC Annexin V−, PI+; C−+).

Since CTAB induced a pronounced dose-dependent decrease in BJ cells’ viability, we decided to check whether eluates of the tested dental composites can induce apoptotic cell death (Figure 2 and Supplementary Table S2) using FC. The obtained results confirmed that, among the tested compounds, only eluates of CTAB -modified composite induced a markedly decrease in the viability of BJ cells (*p* < 0.05). Comparing the percentages of BJ cells in early apoptosis to the percentages in late apoptosis or necrosis after exposure to eluates of the tested compounds, one can observe a dominant late apoptosis or necrosis. Exposure to CTAB eluates and the eluate of 1 wt% DODAB resulted in a statistically significant increase in the percentage of cells in the late apoptotic stage compared to the untreated control group (*p* < 0.05). Furthermore, the eluate of 1 wt% CTAB significantly elevated the proportion of late apoptotic cells when compared to the eluate of 0.5 wt% DODAB (*p* < 0.05) and the eluate of the unmodified KE control composite (*p* < 0.05). A markedly rise in late apoptotic cells was also observed following treatment with the eluate of 1 wt% DODAB when compared to the eluate of 0.5 wt% DODAB (*p* < 0.05). Regarding cell death, treatment with the eluate of 0.5 wt% DODAB induced a significant decrease in the percentage of necrotic cells (*p* < 0.01 vs. control). A higher percentage of necrotic cells was observed in the eluate of 0.5 wt% CTAB (*p* < 0.05) and the eluate of 2.0 wt% (*p* < 0.05) DODAB compared to the eluate of 0.5 wt% DODAB. The eluates of 2 wt% DODAB and the commercial FA composite also showed an elevated number of necrotic cells when compared to the eluate of 0.5 wt% DODAB (*p* < 0.05). Therefore, we can conclude that CTAB-induced cell death was evoked by apoptosis or necrosis.

### 3.3. The Genotoxic Potential of Tested Dental Composites in BJ Cells

**Figure 3:**
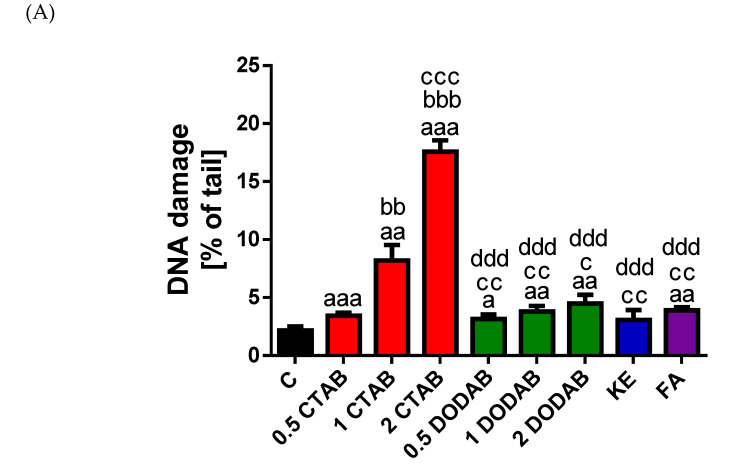

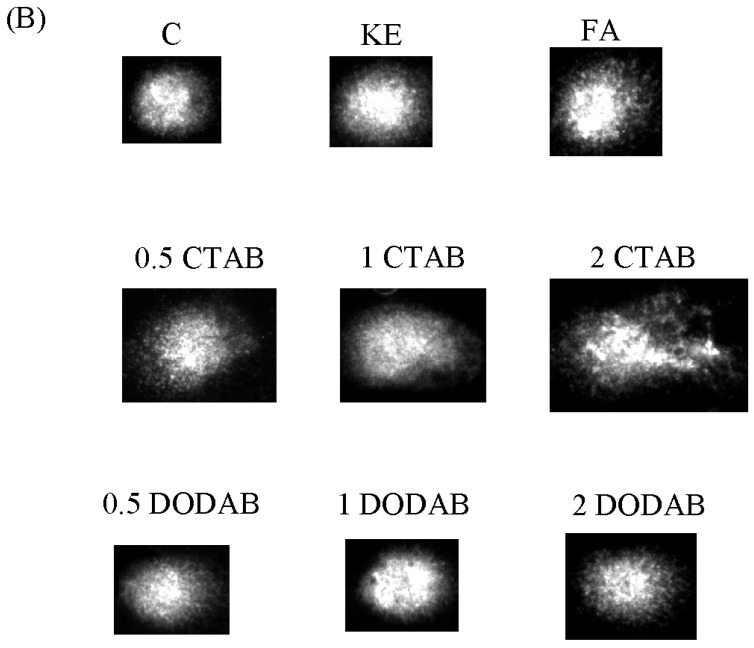
Genotoxic effects of composite eluates on BJ cells. DNA damage was quantified using the alkaline comet assay following 24 h exposure of BJ cells to composite eluates. Treatment included composites modified with CTAB (0.5; 1; 2 wt%), DODAB (0.5; 1; 2 wt%), the unmodified control composite, KE and the commercial composite, FA. (**A**) Quantification of DNA damage: the level of DNA damage in BJ cells exposed to CTAB (0.5%; 1%; 2%; red bars), DODAB (0.5%; 1%; 2%; green bars), KE (blue bar), FA (violet bar), expressed as the percentage of DNA in the comet tail. Data are presented as the mean ± standard deviation (SD). ^a^ *p* < 0.05; ^aa^ *p* < 0.01, ^aaa^ *p* < 0.001 vs. Control (non-treated cells). ^bb^ *p* < 0.01, ^bbb^ *p* < 0.001 vs. 0.5% CTAB. ^c^ *p* < 0.05; ^cc^ *p* < 0.01, ^ccc^ *p* < 0.001 vs. 1% CTAB. ^ddd^ *p* < 0.001 vs. 2% CTAB. (**B**) Representative images of comets after treatment with eluate of composites with CTAB (0.5; 1; 2 wt%), DODAB (0.5; 1; 2 wt%), KE and FA. DNA was visualized by staining with 2 mg/mL 4′,6-diamidino-2-phenylindole dihydrochloride (DAPI). Images were captured at 200× magnification using a fluorescence microscope (Nikon, Tokyo, Japan).

The genotoxic potential of the eluates derived from the tested dental composites on BJ cells was assessed using the alkaline comet assay (Figure 3 and Supplementary Table S3). As anticipated, the eluates of CTAB-modified composite induced a significant dose-dependent increase in DNA damage level compared to the control group. Specifically, all three concentrations demonstrated pronounced damage (0.5 wt% CTAB vs. control, *p* < 0.01; 1 wt% CTAB vs. control, *p* < 0.01; 2 wt% CTAB vs. control, *p* < 0.001). Furthermore, statistical differences were observed among the concentrations themselves (0.5 wt% CTAB vs. 1wt% CTAB, *p* < 0.01; 0.5 wt% CTAB vs. 2 wt% CTAB, *p* < 0.001; 1 wt% CTAB vs. 2 wt% CTAB, *p* < 0.001). Conversely, eluates of the DODAB-modified composite caused only a slight, yet dose-dependent, elevation in the DNA damage level (0.5 wt% DODAB vs. control, *p* < 0.05; 1 wt% DODAB vs. control, *p* < 0.01; 2 wt% DODAB vs. control, *p* < 0.01). However, the eluates derived from all tested concentrations of DODAB induced significantly lower DNA damage compared to the CTAB-modified group. Specifically, DNA damage was lower across all DODAB concentrations when compared to the eluate of 1 wt% CTAB composite (0.5 wt% DODAB vs. 1 wt% CTAB, *p* < 0.01; 1 wt% DODAB vs. 1 wt% CTAB, *p* < 0.01; 2 wt% DODAB vs. 1 wt% CTAB. Furthermore, all DODAB concentration eluates showed highly significant reductions in DNA damage when contrasted with the eluate of 2 wt% CTAB group (*p* < 0.001 for all comparisons). Exposure to the eluate of the unmodified control composite, KE did not induce any measurable change in the level of DNA damage, while the eluate of the commercial composite, FA elicited a minimal increase as compared to control (*p* < 0.01). Eluates derived from the unmodified control composite, KE and the commercial composite, FA consistently induced lower levels of DNA damage compared to the eluates of CTAB-modified groups (vs. 1 wt% CTAB, *p* < 0.01; vs. 2 wt% CTAB, *p* < 0.001).

Taken together, these results demonstrate that CTAB was the only material tested to exert a pronounced genotoxic effect on BJ cells. Importantly, this dose-dependent increase in genotoxicity was directly correlated with the decline in cell viability observed in both the MTT and FC assays.

## 4. Discussion

### 4.1. Cytotoxicity

The obtained results demonstrate that eluates derived from the CTAB-modified composites exhibited the highest cytotoxic potential among all tested material leachates, demonstrating a marked dose-dependent effect. Conversely, eluates from the composites incorporating DODAB, the unmodified control, KE, and the commercial material, FA did not induce a statistically significant reduction in BJ cell viability. This investigation is the first, to our best knowledge, to specifically evaluate the cytotoxic potential RBCs composites incorporating CTAB and DODAB components. A substantial body of literature has documented the pronounced effects of CTAB on cell viability. Zhang et al. [79] reported a significant reduction in the viability of human epidermal keratinocyte (HaCaT) and lung fibroblast (CRL-1490) cells following exposure to 100 µM CTAB at both 2 h and 24 h incubation time points. Further analysis showed that HaCaT cells exhibited no marked decrease in viability when treated with 3 µM and 10 µM CTAB. However, CTAB concentrations exceeding 10 µM induced a statistically significant, dose-dependent decrease in HaCaT cell viability, with the effect being particularly pronounced after 24 h [79]. Pan et al. [80] demonstrated that CTAB concentrations below 12.5 µg/mL elicited a significant reduction in the viability of the hepatocellular carcinoma cell line (HepG2, Hep3B, and Bel-7402) and the normal human liver cell line (HL-7702). A selective cytotoxic effect was observed against the cancer HepG2 line compared to the normal HL-7702 line within the CTAB concentration range of 0.39 µg/mL to 6.25 µg/mL. This cancer cell selectivity was lost when CTAB concentrations exceeded approximately 12.5 µg/mL, suggesting that higher doses induce indiscriminate generalized toxicity affecting both normal and cancer cells [80]. Da et al. [81] reported that CTAB induced a significant time- and dose-dependent reduction in the viability of human osteosarcoma cell lines (HOS, MG63, and U2OS). Conversely, CTAB (0–8 µM) did not significantly affect the viability of the normal human osteoblast cell line (hFOB1.19) [81]. Wu et al. reported that the viability of SK-HEP-1 human hepatic adenocarcinoma cells was maintained above 90% following a 24 h exposure to CTAB (0–5 µM) [82]. A separate study demonstrated that CTAB (0–100 µM) resulted in a concentration-dependent decrease in cell viability across both a panel of six cancer cell lines (FaDu, C-666-1, UTSCC-8A, UTSCC-42A, A549, MCF-7) and two normal cell lines (GM05757 and MRC5), indicating a broad cytotoxic effect within this dose range [83]. Several studies have also evaluated the impact of DODAB on cell viability. Silva et al. [84] characterized DODAB-based liposomes as having minimal cytotoxicity toward mammalian cells, such as the 293T line, at concentrations employed for non-viral gene delivery. In both its capacity as a component of non-viral gene delivery systems and as an antimicrobial agent, DODAB exhibits significantly lower intrinsic toxicity to mammalian cells when compared to other cationic surfactants, notably CTAB [84]. Furthermore, DODAB imparts substantially lower cytotoxicity to the resulting particles than CTAB, an effect demonstrated in human lung fibroblast (SV-80) and human breast adenocarcinoma (MCF-7) cell line following a 48 h exposure period [85]. The cytotoxicity of monomers used in our experimental resin matrix, such as BisGMA, UDMA, TEGDMA, and HEMA has been thoroughly evaluated in previous studies. The literature establishes a consistent hierarchy of toxicity: BisGMA > UDMA > TEGDMA > HEMA [86,87]. Cytotoxic potential is consistently shown to be a function of the dose, exposure time, specific monomer chemistry, and the inherent sensitivity of the tested cell lines [88,89,90,91,92]. Neves et al. [88] demonstrated that BisGMA, UDMA, and TEGDMA induce viability reduction in human peripheral blood mononuclear cells (PBMCs) by MTT assay. Quantitative analysis established the relative toxicity via the TC_50_ values: BisGMA (TC_50_ = 3161.0 mM, TC20 = 2150.0 μM > UDMA TC50 = 505.0 mM, TC20 = 167.0 μM, > TEGDMA (TC50 = 69.0 mM, TC20 = 50.5 μM) [88]. Furthermore, BisGMA (0.06–1 mM), UDMA (0.05–2 mM), and TEGDMA (2.5–10 mM) reduced mitochondrial activity by up to 95%, 93%, and 93%, respectively [88]. BisGMA (30 μM) and UDMA (100 μM) exhibited high toxicity toward human dental pulp cells [86]. UDMA also caused a 40% reduction in murine macrophages viability at 10 µM [89]. TEGDMA cytotoxicity is highly time-sensitive in human dental pulp cells; while low concentrations were benign [93], 3 mM caused a drop in WST values from a control of 1.237 to 0.518 after 24 h, and drastically to 0.056 after 48 h [94]. HEMA demonstrated dose- and time-dependent anti-proliferative effects in human dental pulp cells and caused 55% cytotoxic effect in murine macrophages at 10 mM [95,96].

To delineate the mode of cell death induced by eluates derived from QAS-containing RBCs, we performed flow cytometry analysis. Our results indicate that eluates derived from CTAB incorporating RBCs primarily caused BJ cell death through apoptosis and/or necrosis. Conversely, the DODAB-modified composite, the unmodified control, KE and the commercial material, FA induced only a slight, non-significant increase in cells undergoing late apoptosis or necrosis compared to the untreated control. To the best of our knowledge, this is the first study to assess the potential of dental RBCs containing CATB and DODAB to induce apoptosis or necrosis. Literature data have shown that CTAB exhibits potent pro-apoptotic activity in multiple cancer cell lines through the induction of the mitochondrial cell death pathway and the disruption of key survival signaling cascades. Pan et al. have demonstrated that CTAB treatment for 24 h induced a concentration-dependent increase in the apoptotic rate of human hepatocellular carcinoma (HepG2) cells across a range of (1.5–25 µg/mL) [80]. Mechanistic studies confirmed that this effect was mediated by mitochondrial apoptosis through the activation of the AMPK—p53 signaling pathway [80]. The apoptotic effects of CTAB also involve the suppression of PI3K/AKT cell survival pathway. Da et al. observed that CTAB concentrations ranging from 2–6 µM not only inhibited proliferation and induced cell cycle arrest in osteosarcoma cells but also promoted apoptosis by inhibiting the hyperactive PI3K/AKT pathway [81]. Since PI3K-AKT signaling is a central intracellular cascade regulating tumor cell proliferation, survival, and migration [97], its inhibition by CTAB is critical for inducing cell death. Ito et al. identified CTAB as a potential apoptogenic quaternary ammonium compounds with efficacy against head and neck cancer both in vivo and in vitro [83]. Specifically, they reported that CTAB was able to ablate the tumor-forming capacity of FaDu cells and significantly delayed the growth of established tumors [83]. Studies focusing on the role of CTAB as a caping agent for gold nanorods (AuNRs) have elucidated its potential to induce necrosis, particularly via cell membrane disruption and the subsequent release of inflammatory mediators. Jia et al. [98] observed that CTAB, when used to coat AuNRs, is primarily associated with mouse embryonic fibroblast (NIH-3T3) cell necrosis. The proposed mechanism involves membrane damage and organelle disruption, and damage-associated molecular pattern (DAMPs) release. The positive charge of CTAB interacts strongly with negatively charged cell membrane, causing direct membrane damage and high cellular toxicity, which leads to necrotic cell death. CTAB-coated AuNRs can also target intracellular organelles, such as mitochondria, disrupting their integrity and function [98]. Further research by Zhang et al. [99] corroborated these findings, showing that CTAB-capped AuNPs induced damage that selectively activates cell death pathways characterized by markers of necrosis. Their observation indicated the involvement of cathepsin B, a lysosomal cysteine protease known to be released upon lysosomal membrane destabilization and which is frequently linked to the primary induction of necrotic cell death [99]. One scientific report has specifically addressed the capacity of DODAB to induce apoptosis. Kusumoto et al. [100] demonstrated that DODAB can induce cell death in various tumor cell lines. Specifically, the mechanism of action in human leukemia (HL-60) cells was confirmed to be the induction of apoptosis, involving a complex cascade that includes caspase activation and membrane disruption. A separate, or parallel, proposed mechanism involves the ability of DODAB to induce pore formation in the cell membrane, which may contribute to the overall loss of cellular integrity and subsequent cell death [100]. The effect of monomers used in our experimental resin-matrix has also been studies. Exposure to various dental monomers induces cell death in immune and pulp cells through distinct pathways, often focusing on apoptosis. BisGMA exposure, especially when combined with *Porphyromonas gingivalis*, primarily resulted in necrosis of monocytes [88]. TEGDMA and UDMA predominantly caused cell death via apoptosis [88]. Furthermore, UDMA was shown to induce a dose-dependent shift in murine macrophages: low concentrations triggered early apoptosis, while high concentrations led to late apoptosis and necrosis [89]. HEMA also exhibited dose-dependent cytotoxicity through apoptosis in murine macrophages [95]. TEGDMA induces apoptosis via the activation of multiple caspase cascades. Exposure to TEGDMA of dental pulp cells increased the secretion of apoptosis-induced factor (AIF), which mediates chromatin condensation and DNA degradation. TEGMA differentially activated caspase depending on the concentration: caspase-3 at ≥ 1.5 mM, caspase-8 at 0.1 and 0.2 mM, caspase-9 and caspase-12 at concentrations above 0.75 mM [94]. The activation of caspase -3, -8 and -9 was similarly observed in murine macrophages exposed to 3 mM TEGDMA [101], confirming the involvement of both extrinsic and intrinsic apoptotic pathways.

### 4.2. Genotoxicity

Our findings indicate that only eluates derived from the CTAB-modified composite exerted a significant, dose-dependent genotoxic effect on BJ cells. This increase in DNA damage was inversely correlated with the decrease in cell viability determined by the MTT and FC assay. This study is the first, to our knowledge, to assess the genotoxicity of RBCs containing quaternary ammonium salts. The observed genotoxicity aligns with literature data suggesting that quaternary ammonium salts can induce the production of ROS, leading to oxidative stress [102,103,104]. ROS accumulation and subsequent oxidative stress are known to damage cellular macromolecules, including DNA, lipids, and proteins, often culminating in cytotoxicity, apoptosis, and genotoxicity. The genotoxicity and cytotoxicity induced by RBCs are commonly linked to mitochondrial dysfunction and DNA damage [67,68,86,91,94]. Mitochondria are critical for cellular metabolism and homeostasis [89]. Dysfunction in these organelles triggers further ROS production and depolarization, which mediates caspase-dependent apoptosis [89,95]. Finally, DNA damage itself is characterized by a loss of DNA integrity and strand fragmentation [95].

The cytotoxicity and genotoxicity of dental monomers are primarily driven by their ability to induce oxidative stress through ROS generation and the depletion of glutathione (GSH) reserves [68,105]. Smaller, more hydrophilic monomers like TEGDMA and HEMA can rapidly penetrate cell membrane. Once inside, they disrupt key cellular functions, leading to the suppression of GSH and lipid synthesis, as well as mitochondrial and DNA damage [67,68,86,91,94]. GSH, a critical tripeptide essential for maintaining cellular redox balance, is a central target in monomer toxicity [67,68,91,105]. Depleting cellular GSH compromises the cell’s antioxidant capacity, leading to ROS accumulation, which ultimately damages DNA and induces cell death [67,105]. In fact, GSH detoxification can prevent toxicity even at sub-lethal monomer concentrations [105]. Cysteine is a key amino acid in GSH production. Since cysteine uptake regulates GSH levels, studies have investigated its modulation by different monomers. Short-term (2 h) exposure to 300 μM TEGDMA (300 μM) significantly increased cysteine uptake, while BisGMA and UDMA (300 μM) caused a marked decrease. Long-term (48 h) exposure to BisGMA (<30 μM) and UDMA (<100 μM) significantly reduced cysteine uptake. Importantly, after 48 h, BisGMA and UDMA caused pronounced GSH depletion [86]. TEGDMA is particularly noted for its complex mechanism, which includes generating ROS and inducing the phosphorylation of mitogen-activated protein kinase (MAPKs) [101]. This MAPKs activation is a critical step that impairs biomineralization and drives the activation of apoptosis [67,101].

### 4.3. Clinical Implications

The differential biological safety profiles observed between the modified dental composites lead to clear clinical guidelines for future material development. CTAB-modified composites pose a significant clinical risk. The dose-dependent cytotoxicity, induction of apoptosis/necrosis, and pronounced genotoxicity demonstrated by CTAB-modified composite eluates suggest that these materials have a poor biocompatibility profile. Clinical application of CTAB-modified restoratives could risk irreversible pulpal inflammation and damage to adjacent tissues, likely precluding their direct clinical use in deep restorations. In turn, DODAB-modified composites show superior safety potential. In sharp contrast, eluates from DODAB-modified composites exhibited minimal, statistically non-significant cytotoxicity, apoptosis, and genotoxicity across all tested concentrations up to 2 wt%. This favorable safety profile, consistent with DODAB’s tendency to remain fixed within the material makes it a highly viable and biocompatible candidate for developing safe, next-generation antimicrobial restorative materials that do not compromise host cellular integrity. These results dictate that research efforts should focus on optimizing the DODAB-based composite formulation for antimicrobial function, as the CTAB modification presents an unacceptable biological risk ratio for clinical dental use.

## 5. Conclusions

The combined results demonstrate that only the CTAB-modified composite induced a significant dose-dependent decrease in BJ cell viability, which was concurrent with an increase in DNA damage and cell death primarily attributed to late apoptosis or necrosis. These findings preclude the use of CTAB in dentistry at the concentrations tested due to its clear cytotoxicity and genotoxicity. Conversely, the DODAB-modified composite and the unmodified experimental composite, KE, exhibit favorable biocompatibility profiles that warrant further investigation for potential clinical incorporation into dental materials. Our study has some limitations. A primary limitation of the current investigation is its in vitro design, which necessitates cautious extrapolation of the findings to a complex physiological system. This in vitro study employed a comprehensive replication strategy utilizing three independent biological replicates for each experimental condition, with cells derived from distinct passage numbers. Within each biological replicate, robust technical replication was ensured: 8 replicates for the MTT assay, 50 randomly scored cells for the Comet assay, and 20,000 singlet events acquired for flow cytometry. This design, combining independent replication with high technical fidelity, allows for reliable determination of statistical significance and enhances the generalizability of the findings. In summary, the favorable biological profile of DODAB-modified composites suggests a promising potential for their future application in developing high biocompatible, durable, and intrinsically antimicrobial restorative materials for preventive and therapeutic dentistry. Nevertheless, subsequent in vivo investigations are mandatory to fully translate these promising in vitro findings.

## Figures and Tables

**Table 1:** Summary of QACs actually used in dental materials.

Dental Materials	QACs Used in Dental Materials
dental resins	urethane dimethacrylate monomer with two quaternary ammonium groups [48], dimethylaminododecyl methacrylate (DMADDM) [21], quaternary ammonium methacrylate monomers [49]
dental adhesives/primers	DMADDM [50], 12-methacryloylooxydodecylpiridinium bromide (MDPB) [43], methacryloxyethyl cetyl dimethyl ammonium chloride (DMAE-CB) [51]
dental composites	dimethyldioctadecylammonium bromide (DODAB) [23], cetyltrimethylammonium bromide (CTAB), 2-methacryloxyethyl hexadecyl methyl ammonium bromide (MAE-HB) [52], denture-based acrylic resins: Poly 202063A [53]
quaternary ammonium nanofillers and micro-fillers	quaternary ammonium silica (QASi), quaternary ammonium silane-functionalized methacrylate, quaternary ammonium poly(ethylenimine) nanoparticles (QPEI) [43,54,55,56].

## Data Availability

The original contributions presented in this study are included in this article. Further inquiries can be directed to the corresponding author.

## Supplementary Materials

The following supporting information can be downloaded at: https://www.mdpi.com/article/10.3390/jfb16120459/s1.
