# Supplementary material for: Cytotoxicity and Genotoxicity of Resin-Based Dental Composites Modified with Quaternary Ammonium Salts
Source: J Funct Biomater. 2025 Dec 9;16(12):459. doi: 10.3390/jfb16120459 (PMC12734297; doi:10.3390/jfb16120459)
Supplement: Supplementary file 1 [file jfb-16-00459-s001.zip › jfb-3978982-supplementary.pdf]

Table S1: Effect of composite eluates on BJ cell viability. Data are expressed as the percentage of the untreated control cells (set to 100%), and represent the mean ±standard deviation (SD). $^{a}p < 0.05$; $^{aa}p < 0.01$, $^{aaa}p < 0.001$ vs. Control (non-treated cells). $^{b}p < 0.05$; $^{bb}p < 0.01$, $^{bbb}p < 0.001$ vs. 0.5% CTAB. $^{c}p < 0.05$; $^{cc}p < 0.01$, $^{ccc}p < 0.001$ vs. 1% CTAB. $^{d}p < 0.05$; $^{dd}p < 0.01$, $^{ddd}p < 0.001$ vs. 2% CTAB.

Sample	Cell viability (% of control)
C	100
0.5 CTAB	74.55 ± 11.10 ^{a}
1 CTAB	46.78 ± 5.30 $^{aaa, b}$
2 CTAB	16.93 ± 1.22 $^{aaa, bbb, ccc}$
0.5 DODAB	91.72 ± 4.37 $^{ccc, ddd}$
1 DODAB	89.31 ± 3.10 $^{ccc, ddd}$
2 DODAB	84.64 ± 1.27 $^{ccc, ddd}$
KE	86.67 ± 6.03 $^{ccc, ddd}$
FA	91.09 ± 5.27 $^{ccc, ddd}$

Table S2: Induction of apoptosis in BJ cells by composite eluates. Data are expressed as mean ±standard deviation (SD). $^{a}p < 0.05$; $^{aa}p < 0.01$, $^{aaa}p < 0.001$ vs. Control (non-treated cells). $^{b}p < 0.05$; $^{bb}p < 0.01$, $^{bbb}p < 0.001$ vs. 0.5% CTAB. $^{c}p < 0.05$; $^{cc}p < 0.01$, $^{ccc}p < 0.001$ vs. 1% CTAB. $^{d}p < 0.05$; $^{dd}p < 0.01$, $^{ddd}p < 0.001$ vs. 2% CTAB. $^{e}p < 0.05$; $^{ee}p < 0.01$, $^{eee}p < 0.001$ vs. 0.5% DODAB.

Sample	Live	Early apoptosis	Late apoptosis	Necrosis
C	89.65 ± 1.98	1.15 ± 0.66	6.99 ± 1.38	2.21 ± 0.06
0.5 CTAB	68.95 ± 6.06 ^{a}	1.79 ± 0.58	26.55 ± 5.73 ^{a}	2.71 ± 0.34
1 CTAB	60.04 ± 9.17 ^{a}	4.25 ± 1.13	27.55 ± 5.42 ^{a}	8.17 ± 2.64
2 CTAB	60.77 ± 8.32 ^{a}	1.68 ± 0.26	34.77 ± 8.33 ^{a}	2.78 ± 0.25
0.5 DODAB	86.58 ± 2.12	4.42 ± 1.84	7.79 ± 0.27 $^{b, c, d}$	1.20 ± 0.01 $^{aa, b. d}$
1 DODAB	79.69 ± 3.51	3.23 ± 1.24	12.99 ± 1.24 $^{a, e}$	4.09 ± 1.04
2 DODAB	71.52 ± 8.26	2.32 ± 0.6	23.20 ± 7.21	2.96 ± 0.45 ^{e}
KE	84.89 ± 0.6	3.01 ± 1.26	10.24 ± 0.88 ^{c}	1.85 ± 0.22
FA	79.53 ± 3.08	1.81 ± 0.33	15.74 ± 3.00	2.91 ± 0.41 ^{e}

Table S3: Genotoxic effects of composite eluates on BJ cells. Data are presented as the mean ± standard deviation (SD). $^{a}p < 0.05$; $^{aa}p < 0.01$, $^{aaa}p < 0.001$ vs. Control (non-treated cells). $^{b}p < 0.05$; $^{bb}p < 0.01$, $^{bbb}p < 0.001$ vs. 0.5% CTAB. $^{c}p < 0.05$; $^{cc}p < 0.01$, $^{ccc}p < 0.001$ vs. 1% CTAB. $^{d}p < 0.05$; $^{dd}p < 0.01$, $^{ddd}p < 0.001$ vs. 2% CTAB.

Sample	DNA damage (% of tail)
C	2.15 ± 0.37

0.5 CTAB	3.43 ± 0.27^{aaa}
1 CTAB	$8.20 \pm 1.34^{aa, bb}$
2 CTAB	$17.56 \pm 0.99^{aaa, bbb. ccc}$
0.5 DODAB	$3.16 \pm 0.40^{a, cc, ddd}$
1 DODAB	$3.80 \pm 0.49^{aa, cc, ddd}$
2 DODAB	$4.48 \pm 0.76^{aa, c, ddd}$
KE	$3.07 \pm 0.85^{cc, ddd}$
FA	$3.91 \pm 0.27^{aa, cc, ddd}$
